# Case report: Desquamating dermatitis, bilateral cerebellar lesions in a late-onset methylmalonic acidemia patient

**DOI:** 10.3389/fneur.2023.1255128

**Published:** 2023-09-22

**Authors:** Qihua Chen, Jianguang Tang, Hainan Zhang, Lixia Qin

**Affiliations:** Department of Neurology, The Second Xiangya Hospital, Central South University, Changsha, Hunan, China

**Keywords:** desquamating dermatitis, cerebellum, genes, cobalamin C deficiency, skin lesions

## Abstract

**Introduction:**

Cobalamin C (cblC) deficiency is a rare hereditary disorder affecting intracellular cobalamin metabolism, primarily caused by mutations in *MMACHC*. This condition is characterized by combined methylmalonic acidemia and hyperhomocysteinemia, displaying a wide range of clinical manifestations involving multiple organs. Owing to its uncommon occurrence and diverse clinical phenotypes, diagnosing cblC deficiency is challenging and often leads to delayed or missed diagnoses.

**Case description:**

In this report, we present a case of late-onset cblC deficiency with brown desquamating dermatitis on the buttocks. Magnetic resonance imaging (MRI) of the brain revealed bilateral cerebellar abnormalities. The suspicion of an inherited metabolic disorder was raised by abnormal serum amino acid and acylcarnitine levels, along with increased urine methylmalonic acid and serum homocysteine levels. Whole-exome sequencing helped identify a homozygous variant (c.482G>A) in *MMACHC*, confirming the diagnosis of cblC deficiency. However, despite receiving treatment with hydroxocobalamin and betaine, the patient did not experience clinical improvement, which may be attributed to the delayed diagnosis as indicated by the declining homocysteine and methylmalonic acid levels.

**Conclusion:**

Collectively, we emphasize the significance of recognizing the skin lesions and observing serial MRI changes in patients with cblC deficiency. Our case underscores the importance of early diagnosis and timely therapeutic intervention for this severe yet frequently manageable condition.

## Introduction

Combined with methylmalonic acidemia and homocystinuria, cobalamin C (cblC) is the most common inborn error in cobalamin metabolism. Newborn genetic screening studies for metabolic errors revealed that the estimated incidence of CblC deficiency is 1/3,920 in Shandong Province, China (1), and 1/100,000 in New York, United States (2). In 2006, Lerner-Ellis et al. (3) cloned the disease-causing *MMACHC* gene. To date, over 50 mutations have been identified in patients with *MMACHC*. Mutations in *MMACHC* disrupt the intracellular metabolism of cobalamin, causing the accumulation of methylmalonic acid and homocysteine and a lack of methionine. However, the underlying pathophysiological mechanisms are not well understood.

cblC deficiency is associated with systemic involvement and complex clinical manifestations, including progressive encephalopathy, subacute combined degeneration of the spinal cord, maculopathy, anemia, thromboembolic complications, pulmonary arterial hypertension, and kidney injury (4, 5). Skin manifestations, although rare, are often overlooked (6). Brain magnetic resonance imaging (MRI) findings commonly include hydrocephalus, progressive white matter abnormalities, and atypical lesions in the basal ganglia and cerebellum (4, 7). Late-onset cblC deficiency, which occurs in approximately 10% of affected individuals aged above 12 months, generally presents with milder symptoms, more evident clinical presentations and improvement, and a more favorable prognosis than that observed in the early-onset form. However, some patients do not respond well to treatment, particularly those with severe complications or delayed or insufficient initiation of therapy. In this case report, we describe a 21-year-old man who presented with psychiatric symptoms, skin lesions, and bilateral cerebellar abnormalities on brain MRI. The diagnosis of cblC deficiency was confirmed using whole-exome sequencing.

## Case description

**Figure 1:**
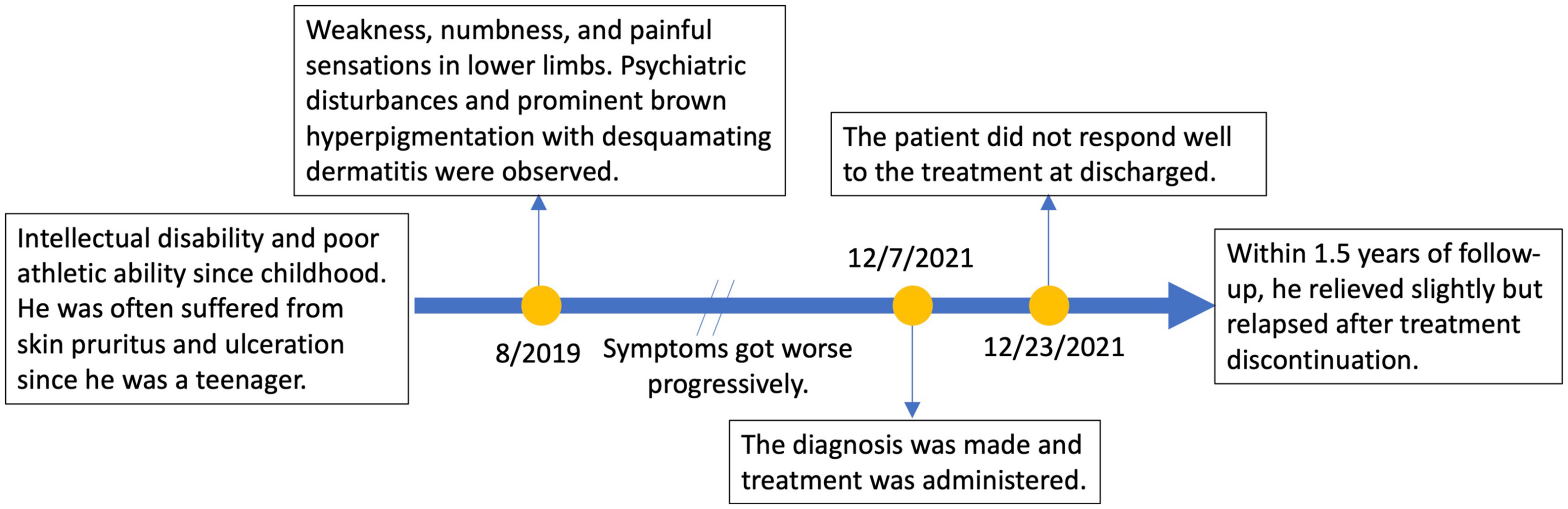
A timeline of events.

**Figure 2:**
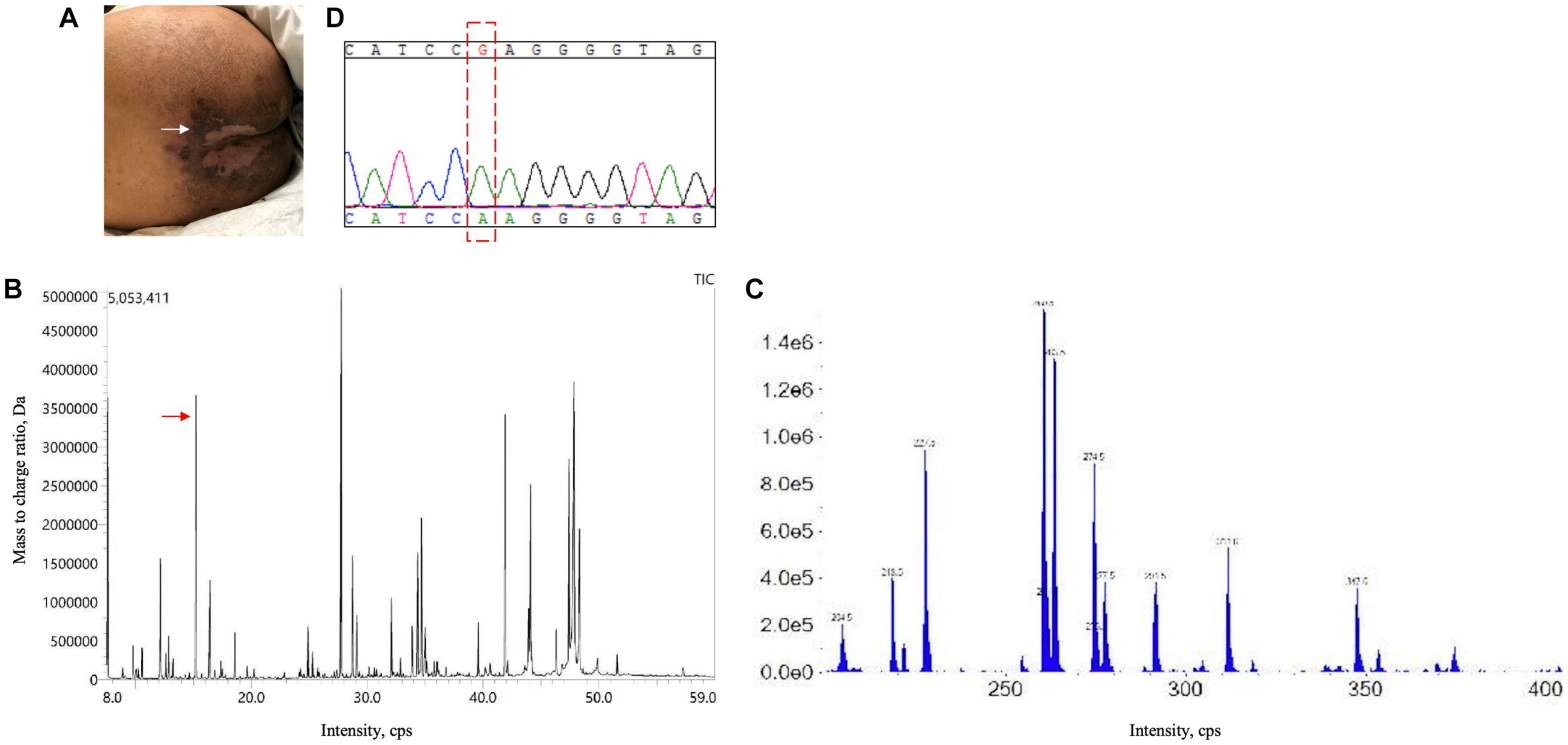
Skin lesion, urine organic acid analysis, serum amino acid and acylcarnitine analysis, and genetic testing results. **(A)** Superficial, erosive desquamating dermatitis on the buttocks of the present patient. **(B)** Urine organic acid analysis revealed an evident increase in methylmalonic acid levels (24.8 μmol/L; normal range: <4.0 μmol/L). **(C)** Serum amino acid and acylcarnitine analysis exhibited an increased propionyl carnitine/acetylcarnitine ratio (0.33; normal range: 0.02–0.2). **(D)** Next-generation sequencing identified a homozygous variant (c.482 G > A) in *MMACHC*, which was further validated by sanger sequencing.

**Figure 3:**
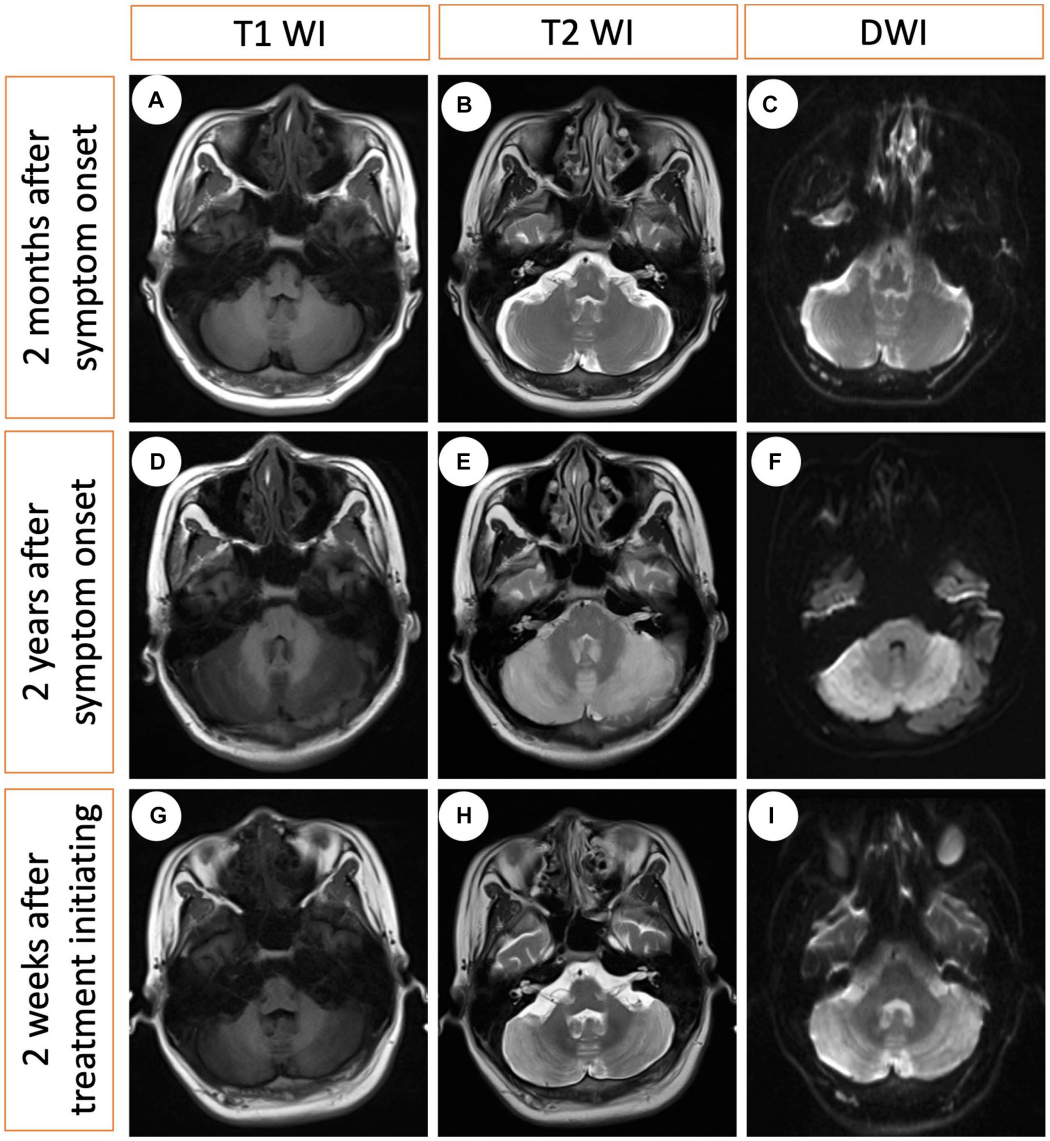
Serial MRI changes at three different time points. **(A–C)** The first MRI obtained 2 months after symptom onset (no treatment), indicating modest cerebral atrophy. **(D–F)** The second MRI obtained 2 years after symptom onset (no treatment), revealing bilateral hyperintensity in the cerebellum on the T2-weighted image and diffusion-weighted imaging (DWI), with hypointensity on the T1-weighted image. **(G–I)** The third MRI obtained 2 weeks after treatment initiation indicating minimal changes.

A 21-year-old man with no significant family history presented with a two-year history of weakness, numbness, and painful sensations in his lower limbs. Over the course of 1 month, he also experienced psychiatric disturbances. Figure 1 provides a timeline of the events. The patient reported additional symptoms, including urinary and bowel incontinence, as well as intellectual and motor developmental delays. He exhibited a motionless posture with a distressed facial expression, occasionally crying out loudly. Seborrheic dermatitis was observed on his face and trunk, and the buttocks had prominent brown hyperpigmentation with desquamating dermatitis (Figure 2A). Tendon reflexes were normal, with no signs of pyramidal neurological deficits. However, the patient’s impaired consciousness hindered further examination. Laboratory tests revealed hypochromic microcytic anemia (hemoglobin level: 91 g/L), elevated transaminase levels (glutamic-pyruvic transaminase: 168.7 U/L; aspartate aminotransferase: 84.1 U/L), and significantly increased serum homocysteine levels (258.4 μmol/L, normal range, 5–15.0 μmol/L). Urine organic acid analysis and serum amino acid and acylcarnitine analysis showed substantially elevated urine methylmalonic acid levels (24.8 μmol/L, normal range, <4.0 μmol/L) and serum propionyl carnitine/acetylcarnitine ratio (0.33, normal range, 0.02–0.2) (Figures 2B,C). No signs of infection, inflammation, or toxicity were present, and serum and cerebrospinal fluid (CSF) anti-cerebellitis antibodies were negative. Spinal cord MRI scan results were normal. Electromyogram (EMG) findings indicated widespread motor and sensory demyelination with accompanying axonal injuries in the limbs, particularly affecting the lower limbs. The first MRI, obtained 2 months after symptom onset, revealed diffuse cerebral atrophy (Figures 3A–C). Subsequent MRI performed at this visit (2 years after symptom onset) revealed bilateral symmetrical lesions in the cerebellum, characterized by hyperintensity on T2-weighted images, restricted diffusion on diffusion-weighted imaging (DWI), and no enhancement (Figures 3D–F). Based on the abnormal serum amino acid and acylcarnitine levels, elevated urine methylmalonic acid level, and increased serum homocysteine level, inherited metabolic diseases were strongly suspected. The diagnosis of late-onset cblC deficiency was further supported by whole-exome genetic sequencing, revealing a homozygous variant (c.482 G > A) in *MMACHC* (Figure 2D). Further genetic analysis proved that the patient in our case was maternal uniparental disomy (mUPD). Following treatment with adequate betaine (9 g/day, administered intragastrically), cobamamide (1.5 mg/day, administered via intramuscular injection), mecobalamin (1 mg/day, administered via intramuscular injection), and B vitamins, the patient’s serum homocysteine levels substantially decreased (60.9 μmol/L), as did urine methylmalonic acid levels (12.5 μmol/L). At discharge, the patient did not experience significant clinical improvement, as well as observed changes on brain MRI (Figures 3G–I). Within 1.5 years of follow-up, he relieved slightly but relapsed after treatment discontinuation.

## Discussion

cblC deficiency is the most prevalent disorder associated with cobalamin metabolism (5) and is a rare autosomal recessive disease induced by homozygous recessive or compound heterozygous mutations in *MMACHC* (3, 8). These mutations disrupt cobalamin metabolism, leading to the accumulation of toxic levels of metabolites, homocysteine and methylmalonic acid, which cause oxidative injury. While the central and peripheral nervous system are commonly affected, cblC deficiency can also involve more organs and tissues, including the kidneys (9), lung (10), micrangium (11), retina (12), and skin (6). Early-onset cblC deficiency, with an age of onset <12 months, is more prevalent and typically manifests with feeding difficulties, somnolence, developmental delays, seizures, muscular hypotonia, hydrocephalus, and microcephaly (1, 4, 8, 13). Late-onset cblC deficiency is characterized by neuropsychiatric symptoms, cognitive dysfunction, myelopathy, rental function abnormality, and pulmonary arterial hypertension (14, 15). The present case exhibited psychiatric symptoms, cognitive decline, peripheral neuropathy, dermatitis, anemia, and hepatic dysfunction were found.

Diffuse cerebral atrophy and bilateral hyperintensity in the deep white matter and cerebellum are common brain MRI findings in patients with late-onset cblC deficiency (15, 16). The patient presented with symmetrical bilateral cerebellar lesions. The differential diagnoses of cerebellar lesions are broad and include vascular causes (occlusion of lateral posterior inferior cerebellar artery), infectious agents (such as bacteria, viruses, and mycoplasma), autoimmune factors (such as anti-glutamate decarboxylase 65 antibody-associated cerebellitis, paraneoplastic), hereditary conditions (hereditary spinocerebellar ataxia, fragile X–associated tremor ataxia syndrome), as well as metabolic and toxic factors (such as alcohol, cytotoxicity drugs, organic solvent) (17–20). The clinical history and laboratory analyzes narrowed the diagnostic possibilities, and genetic testing confirmed the final diagnosis.

Skin lesions owing to nutritional deficiency are rare manifestations of extra-nervous system involvement in cblC deficiency. They can present as cheilitis, diffuse erythema with erosions and desquamation, and superficial erosive desquamating dermatitis, which may be attributed to enzymatic deficiency or nutritional restriction (6, 21, 22). Researchers have suggested that erosive desquamating dermatitis with histopathological characteristics resembling acrodermatitis enteropathica may be an initial systemic sign of cblC deficiency. The presence of brown desquamation dermatitis should prompt clinicians to consider this diagnosis when nutritional deficiency is suspected. Renal examination was normal, but screening for optic neuropathy and retina was not performed for the patient’s poor psychiatric status.

CblC deficiency is often curable. Most patients with late-onset cblC deficiency respond well to hydroxocobalamin and betaine treatment. The goals of treatment are to improve clinical presentation, normalize serum methionine levels, reduce urine methylmalonic acid levels, and lower homocysteine levels by promoting homocysteine-to-methionine conversion, facilitating re-methylation, and accelerating acylcarnitine clearance (5, 23). The patient in our case only received betaine, cobamamide, and mecobalamin, not hydroxycobalamin, because hydroxycobalamin injection was not available in many provinces of China. Delayed diagnosis and therapeutic intervention, low compliance to maintenance treatment, and no hydroxycobalamin was given contributed to the poor outcomes in this case. Constant and aggressive treatment seems to be required.

In conclusion, we reported a rare case of late-onset cblC deficiency with a delayed diagnosis, presenting with brown desquamation dermatitis and bilateral cerebellar MRI abnormalities. Owing to its rarity and heterogeneous clinical presentation, the diagnosis of cblC deficiency poses challenges and is often delayed or missed. Our case highlights the importance of early diagnosis and timely therapeutic intervention for this severe but often treatable disease. Otherwise, irreversible damage to multiple organs, particularly neurological complications, is unavoidable, ultimately resulting in poor outcomes.

## Data availability statement

The datasets presented in this article are not readily available because of ethical and privacy restrictions. Requests to access the datasets should be directed to the corresponding author.

## Ethics statement

The studies involving human participants were reviewed and approved by the Ethics Committee of the Second Xiangya Hospital, Central South University. The patients/participants provided their written informed consent to participate in this study. Written informed consent was obtained from the individual(s) for the publication of any potentially identifiable images or data included in this article.

## Author contributions

QC: Writing – original draft, Writing – review & editing. JT: Writing – original draft, Writing – review & editing. HZ: Writing – original draft, Writing – review & editing. LQ: Writing – original draft, Writing – review & editing.

## Funding

This work was supported by grant from the National Natural Science Foundation of China (No. 82101342 to LQ), the Provincial Natural Science Foundation (No. 2022JJ30833 to LQ), and Scientific Research Launch Project for new employees of the Second Xiangya Hospital of Central South University.

## Conflict of interest

The authors declare that the research was conducted in the absence of any commercial or financial relationships that could be construed as a potential conflict of interest.

## Publisher’s note

All claims expressed in this article are solely those of the authors and do not necessarily represent those of their affiliated organizations, or those of the publisher, the editors and the reviewers. Any product that may be evaluated in this article, or claim that may be made by its manufacturer, is not guaranteed or endorsed by the publisher.
